# Measuring Alliance and Symptom Severity in Psychotherapy Transcripts Using Bert Topic Modeling

**DOI:** 10.1007/s10488-024-01356-4

**Published:** 2024-03-29

**Authors:** Christopher Lalk, Tobias Steinbrenner, Weronika Kania, Alexander Popko, Robin Wester, Jana Schaffrath, Steffen Eberhardt, Brian Schwartz, Wolfgang Lutz, Julian Rubel

**Affiliations:** 1 Department of Psychology, Osnabrück University, Osnabrück, Germany; 2 Department of Psychology, University of Trier, Trier, Germany

**Keywords:** Natural language processing, Computational psychotherapy research, Machine learning, Explainable artificial intelligence

## Abstract

We aim to use topic modeling, an approach for discovering clusters of related words (“topics”), to predict symptom severity and therapeutic alliance in psychotherapy transcripts, while also identifying the most important topics and overarching themes for prediction. We analyzed 552 psychotherapy transcripts from 124 patients. Using BERTopic (Grootendorst, 2022), we extracted 250 topics each for patient and therapist speech. These topics were used to predict symptom severity and alliance with various competing machine-learning methods. Sensitivity analyses were calculated for a model based on 50 topics, LDA-based topic modeling, and a bigram model. Additionally, we grouped topics into themes using qualitative analysis and identified key topics and themes with eXplainable Artificial Intelligence (XAI). Symptom severity could be predicted with highest accuracy by patient topics ($r$=0.45, 95%-CI 0.40, 0.51), whereas alliance was better predicted by therapist topics ($r$=0.20, 95%-CI 0.16, 0.24). Drivers for symptom severity were themes related to health and negative experiences. Lower alliance was correlated with various themes, especially psychotherapy framework, income, and everyday life. This analysis shows the potential of using topic modeling in psychotherapy research allowing to predict several treatment-relevant metrics with reasonable accuracy. Further, the use of XAI allows for an analysis of the individual predictive value of topics and themes. Limitations entail heterogeneity across different topic modeling hyperparameters and a relatively small sample size.

**Supplementary Information:**

The online version contains supplementary material available at 10.1007/s10488-024-01356-4.

## Introduction

After decades of psychotherapy outcome research, the effectiveness of psychotherapy has been convincingly shown in numerous clinical and naturalistic studies and meta-analyses across a broad range of different conditions (for an overview, see Lambert, 2021). More recently, the focus has shifted to the processes and mechanisms of change that drive the effectiveness of psychotherapy (Lutz et al., 2021). At the core, process research tries to identify ingredients and mechanisms that either allow psychological interventions to work or increase their desired effects. Several processes have been identified, most prominently the alliance, but also many others (see Crits-Christoph et al., 2021). The effects and clinical utility of these processes have been shown in a landmark meta-analysis by Norcross and Lambert (2019). However, limitations prevail, such as unknown directions of the process–outcome relationship (e.g., is the alliance driving improvements in outcome or the other way around?) and difficulties to distinguish patient and therapist effects. Another limitation is a lack of research on what could be called second-order processes. Second-order processes relate to processes as processes relate to treatment outcome: They are the ingredients and mechanisms that allow processes to be effective. As such, for example, they aim to answer the important question ‘What ingredients and mechanisms does it take for the intervention to improve the alliance?’ just as processes aim to answer the question ‘What ingredients and mechanisms does it take for the intervention to improve outcome?’. Second-order processes are important, because often, it is far from self-evident how to foster therapeutic processes (Norcross & Lambert, 2019). A final limitation of process research lies in the fact that it is often focused on questionnaire data for large analyses as qualitative assessments can be very time-consuming. This leads to several problems: First, questionnaire data must be filled by patients, which can be both time-consuming and burdensome. Second, questionnaires are better suited for a top-down approach, since they must be relatively short so that a specific construct needs to be pre-selected. If we want to conduct a more bottom-up (i.e., data-driven) analysis, they fall short due to a lack of items. Third, patients may not quite understand the items the way they were intended (e.g., Hawkins et al., 2017). Instead, using Natural Language Processing (NLP), we can use therapy transcripts to create features that predict relevant constructs. This allows for a more data-driven analysis, employing the patient’s own words across the whole session without resorting to session-wise questionnaires with few items that may become burdensome for the patient. We aim to exemplify this approach by using the session content to predict treatment progress (i.e., symptom severity) and a fundamental mechanism (i.e., alliance) building on prior work (e.g., Aafjes-van Doorn et al., 2020; Burkhardt et al., 2022; Goldberg et al., 2020; Ryu et al., 2023). By analyzing transcript features we aim to identify first-order processes that correlate with outcome and second-order processes that correlate with the alliance. We hope that this approach may enrich process research by providing new opportunities to analyze different processes and their respective interactions.

The value of session-wise assessments of symptom severity and alliance can also be viewed through the lens of routine outcome monitoring (ROM) and patient-focused research (Castonguay et al., 2013). Based on research that has proven clinical intuition to be inaccurate (Ægisdóttir et al., 2006), data-driven prognostic models have been developed to predict treatment outcome for specific patients. These models rely on ROM by measuring symptom severity on a session-to-session level since symptom severity is a robust predictor of therapy outcome (e.g., Lorenzo-Luaces et al., 2020). By employing ROM and giving psychometric feedback to the therapist, patient-focused research seeks to increase therapists’ awareness of their patient’s progress or lack thereof. Feedback can be more or less statistically and methodologically sophisticated, from raw outcome scores to expected treatment response (ETR) curves with additional clinical support tools (e.g., Lutz et al., 2022; Whipple et al., 2003). In several meta-analyses feedback has shown additional treatment effects beyond the mere intervention, with further benefits regarding treatment duration and dropout rates (for an overview, see Lutz et al., 2021). Even though the exact mechanisms of feedback remain unknown, it is hypothesized that it may work by increasing therapists’ awareness of clients in risk of deterioration and supporting the therapists with tools to counter these developments early on (e.g., Delgadillo et al., 2018). Also, it remains unknown whether the effects of ROM feedback could possibly also apply to some degree to alliance feedback as the alliance is one of the most investigated psychotherapy processes and a strong indicator of good outcome (*r =* .28; Flückiger et al., 2018). According to Bordin’s (1979) pantheoretical definition, the working alliance entails three components: Consensus on therapeutic goals, agreement on the tasks that lead to these goals, and the affective bond between therapist and patient. Similar to symptom severity, a within-person and between-person distinction can be made. Accordingly, the alliance can be conceptualized both as a general factor that mediates the effects of baseline characteristics on outcome and an idiosyncratic process that evolves over the course of therapy (Zilcha-Mano & Fisher, 2022). This distinction has proven fruitful as the alliance has been measured on a session-to-session level to predict symptom change over the course of therapy (e.g., Rubel et al., 2017).

With advancements in artificial intelligence and machine learning, researchers now wield powerful tools for analyzing big data (O’Leary, 2013), leading to new approaches in psychotherapy research (Aafjes-van Doorn et al., 2021). However, methodological challenges remain, such as heterogeneity of cross-validation strategies, a broad range of different algorithms without any consensus which one might perform best for a given problem, and limited model interpretability, especially with more complex algorithms. Addressing these challenges may involve employing more robust nested k-fold cross-validation procedures (Tibshirani et al., 2021) and an eXplainable Artificial Intelligence (XAI) framework (Holzinger et al., 2022).

Machine learning approaches from the area of NLP allow for a quantification and a subsequent statistical analysis of language. This applies for unsupervised algorithms, which can be used for clustering (e.g., PCA, DBSCAN), allowing for complexity reduction in large data sets. One promising approach is topic modeling (Kherwa & Bansal, 2018). Topic modeling is a set of statistical and probabilistic techniques to detect clusters of related words (“topics”) in a collection of documents. Popular techniques entail Latent Semantic Analysis (LSA; Deerwester et al., 1990), Non-negative Matrix Factorization (NMF; Paatero & Tapper, 1994) and the Latent Dirichlet Allocation (LDA; Blei et al., 2003). These techniques are based on bag-of-words-embeddings that measure the frequency of words in the respective document but ignore relevant information such as word order, textual context, or semantic similarities. Yet, they have been successfully implemented in many areas of research (Kherwa & Bansal, 2018) including psychotherapy research. Atkins et al. (2012) conducted topic modeling on a corpus from a couples therapy trial showing two important benefits: Topic modeling can be used to predict relevant target variables, such as symptom load or alliance. Also, it can be used to analyze therapeutic processes by looking at the upcoming topics. Further, Atkins et al. (2014) successfully predicted the treatment fidelity in sessions of motivational interviewing with labeled topic modeling, a topic modeling approach to classify documents according to their respective label. Imel et al. (2015) showed that topic modeling creates highly relevant topics in individual therapies. Using labeled topic modeling, they were able to discriminate different therapy approaches. Atzil-Slonim et al. (2021) found that topics accurately reflected the psychotherapeutic process. They were able to cluster topics in superordinate themes that comprised a broad perspective of therapy-related topics. Further, they could predict good outcomes and the occurrence of alliance ruptures in a logistic regression while simultaneously identifying associated topics.

Recently, the rise of deep learning allowed for the development of neural topic modeling, which shows superior performance to the “classic” approaches mentioned above (Miao et al., 2016). This is due to their more complex and accurate embeddings taking into account word similarities, word order, and textual context. Lin et al. (2022) compared several neural topic modeling approaches showing their potential to monitor the therapeutic process. More recently, deep learning transformer embeddings have shown even better performance (Vaswani et al., 2017), leading to the development of a topic modeling approach based on transformer embeddings (BERTopic; Grootendorst, 2022).

## Objectives

This study aims to overcome some of the aforementioned limitations by using BERTopic topic modeling in a nested cross-validation procedure to compare several promising machine learning algorithms to predict symptom severity and alliance. Moreover, we want to employ XAI to identify both important topics as well as important superordinate topic themes for each of the predictions. This will allow us to gain insight into session processes that drive change in symptom severity and alliance. Further, we would like to provide a proof of concept for future patient-focused and routine outcome monitoring research: By using NLP and XAI, feedback may be provided solely based on the session transcript, highlighting the driving processes for the prediction.

## Methods

### Patients and Therapists

The sample consisted of 124 patients treated at an outpatient clinic in Trier, Germany. Most patients suffered from a primary diagnosis of depression. Diagnosis was determined with the Structured Clinical Interview for Axis I DSM-IV Disorders-Patient Edition (SCID-I; First & Gibbon, 2004). Patients were mostly diagnosed with a primary diagnosis of recurrent depressive disorder (*n* = 33), major depressive disorder (*n* = 14), or dysthymia (*n* = 9). Other frequent primary diagnoses included adjustment disorder (*n* = 10), social anxiety disorder (*n* = 8), agoraphobia (*n* = 8), PTBS (*n* = 6), or OCD (*n* = 6). They were diagnosed with up to three comorbid disorders, mainly depressive disorders or dysthymia (*n* = 84), anxiety disorders (*n* = 59), adjustment or trauma related disorders (*n* = 26). Further, 15 patients were diagnosed with an eating disorder, 8 patients were diagnosed with a personality disorder and 52 patients were diagnosed with other additional disorders.

Treatment was delivered by 47 therapists. All therapists had at least one year of prior clinical experience. They had finished a Master’s degree in clinical psychology and were either currently doing their CBT psychotherapy training or were already licensed CBT psychotherapists. Even though CBT disorder-specific treatments were applied, therapists also integrated interpersonal and emotion-focused techniques.

Most therapists were treating only one or two patients in this sample (*mean* = 2.57, *sd* = 2.64). The number of patients treated per therapist ranged from 1 up to 14 patients. All therapists received regularly supervision during the course of the study. Therapy sessions were videotaped for supervision and research purposes.

### Treatment

Patients received weekly sessions of integrative CBT (CBT including third-wave and interpersonal and emotion-focused approaches). Outcome data was routinely collected prior to each session and process data was collected for therapist and client after each session. Treatments consisted on average of 35.74 sessions (*sd* = 19.74). The treatment by the attending therapist started in the third session, following a comprehensive screening by an experienced colleague in the first session and a clinical interview (SCID-I) in the second session.

### Instruments and Measures

#### Alliance

After each session, the therapeutic alliance was assessed by the Session Rating Scale (SRS; Duncan et al., 2003). The SRS reflects the three pillars of the therapeutic alliance (1. Affective bond, 2. Goal agreement, 3. Task agreement) as proposed by Bordin (1979). The SRS has good internal consistency, with Cronbach’s alpha values ranging from 0.70 to 0.97 in clinical populations (Murphy et al., 2020). Test-retest reliability estimates range from 0.54 to 0.70, indicating some stability over time (Murphy et al., 2020). Regarding concurrent validity, the SRS provides a moderate correlation with the HAQ II (*r* = .48; Duncan et al., 2003) and the WAI (*r* = .57–0.65; Reese et al., 2013). In this particular sample, the SRS showed sufficient internal consistency during the third session, which was the first session included in this analysis ($\omega$=0.83).

### Symptom Severity

Data was gathered to monitor the progress of patients undergoing therapy using the Hopkins Symptom Checklist-short form (HSCL-11; Lutz et al., 2006). This is an 11-item questionnaire that measures the level of psychological distress experienced by patients. Each item asks the patient to rate the extent of their symptoms over the past week using a four-point Likert scale. The HSCL-11 has been found to be highly correlated with other well-established measures of anxiety and depression symptom distress (Lutz, Jong, Lutz et al., 2021a, b), such as the Brief Symptom Inventory (BSI; Derogatis & Melisaratos, 1983; *r* = .91) and its depression (*r* = .91) and anxiety (*r* = .82) subscales, the Patient Health Questionnaire-9 (PHQ-9; Kroenke et al., 2001; *r* = .81), the Beck Depression Inventory-II (BDI-II; Beck et al., 1996; *r* = .70), and the Generalized Anxiety Disorder 7 (GAD-7; Spitzer et al., 2006; *r* = .72). The HSCL-11 has been used successfully in previous studies to monitor patient progress and has shown comparable sensitivity to change as other measures (e.g., Rubel et al., 2017). In this particular sample, the HSCL-11 showed good internal consistency during the third session, which was the first session included in this analysis ($\omega$=0.92).

#### Transcripts

Transcripts were collected from 124 patients. On average, 4.45 (*sd* = 4.86) session transcripts were included per patient leading to 552 transcripts. The transcripts were usually taken from the beginning of the treatment, starting with session 3 and following with every 5th session (e.g., session 3, 5, 10, 15, …). The transcripts begin with the third session, because the first two sessions mainly served a diagnostic function and were conducted by different psychotherapists. From the third session on, the actual treatment begins, which is conducted by the same therapist. After that, due to limited resources, it had been decided that only every fifth session would be transcribed. However, the number of sessions was imbalanced with ten patients with ten or more sessions and 27 patients with only one session included.

The transcripts were created by psychology students based on the session videotapes. In general, transcripts were spelled according to German orthographic guidelines. All transcripts were anonymized to conceal personal information such as names and cities. The transcripts are segmented by speech turns with speaker identification. No time stamps are provided. Nonverbal cues or background noises and interruptions are annotated in parentheses. No transcription software tools were used.

Altogether, there were 104,557 patient speech turns with an average length of 23.7 (*sd* = 29.5) words and 189.4 (*sd* = 83.2) speech turns per session. For therapists, there were 88,345 speech turns with an average length of 18.0 words (*sd* = 20.9) and an average of 161.8 (*sd* = 64.2) speech turns per session.

### Data Analytic Strategies

The analyses were conducted mainly with Python 3.9 (Python Software Foundation, 2023) with some analyses conducted with R 4.2 (2022).

### Topic Modeling Preprocessing

Transcripts were separated by client and therapist speech so that each step was conducted for therapist and client data separately. Further, the transcripts were split into large lists with each speech turn corresponding to one list item. We conducted very little preprocessing, since the BERT-model requires no preprocessing. However, special characters were removed as well as hesitation vowels (e.g., “ehm”) and descriptions of nonverbal actions that were provided in parenthesis by transcribers (e.g., “(laughing)”). Speech turns with less than five words after the preprocessing were eliminated. For the creation of the embeddings the sentence-transformers language model *paraphrase-multilingual-MiniLM-L12-v2* (Reimers & Gurevych, 2019) was used. This model maps sentences and paragraphs on a 384-dimensional vector space and can be used for clustering. Also, the model is trained on a multilingual corpus including German texts and could therefore be used for German transcripts.

### Topic Modeling

The embeddings were then fed to BERTopic, which can be run with several specifications: We selected as tokenizer a count vectorizer that also includes n-grams within the range 1–3 and also the c-TF-IDF weighting scheme. Together, these are responsible to identify the representative words for each topic. The n-gram range allows not only single words to be representative, but also up to three words in a row. We ran BERTopic both for therapist as well as patient embeddings six times each, varying the number of words per list item (5 or 10 words) and the number of generated topics (150, 200, or 250 topics). Two master students with clinical psychology training assessed the topic quality of the generated topics for each of the six models with an interrater-reliability of *ICC* = 0.61. For patient and therapist topics, the model with a minimum word count of 5 and with 250 topics was rated as the model with the highest quality topics and was therefore selected.

### Topic Theme Clustering

Two independent master’s students in clinical psychology were asked to cluster the topics qualitatively according to themes (Hill & Knox, 2021). In a consensus meeting they came to an agreement about the thematic clusters. In case of persistent disagreement, a PhD student in psychotherapy research undergoing psychotherapy training mediated until consensus. Again, therapist and patient topics were clustered separately.

### Machine Learning Preprocessing

BERTopic allows for the approximation of the probability of topic occurrence within each list item of speech turns. The approximation is based on the distance of the dimensionally reduced embedding clusters calculated by HBDSCAN (McInnes & Healy, 2017). These probabilities were aggregated as sums on the session level so that the topic frequency for each session was approximated and so that they could subsequently be used as features in the machine learning algorithm.

### Building and Evaluating Predictive Models

We chose two different target variables: (1) The SRS mean score of the current session as working alliance score and (2) the HSCL-11 mean score before the current session as symptom severity score. The aggregated topic frequencies were selected as the only features in the prediction. In case of missing values in the target variable, the respective data row was eliminated.

**Fig. 1:**
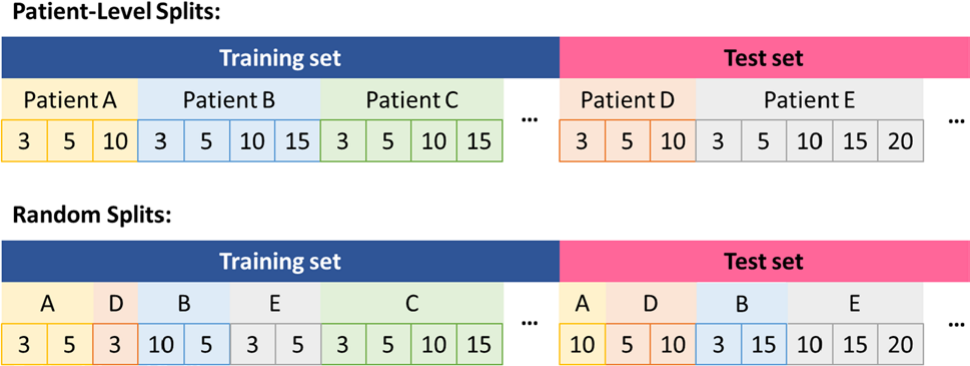
Comparison of patient-level splits and random splits

We employed nested 10-fold cross-validation, splitting the data into ten external folds and using internal five-fold cross-validation for model selection to prevent overfitting. This approach ensures that each external testing fold is predicted only once by the best performing algorithm in the internal folds. We implemented two procedures: random session splits as our main analysis and patient-level splits as sensitivity analysis, the latter to enhance generalizability by keeping sessions from the same patient either in the training or test set but not split across both. (see Fig. 1).

In total, 8 models were generated: Two sets based on patient and therapist topics, each predicting two target variables (alliance, symptom severity) and cross-validation conducted on either random or patient-level splits. Regarding model metrics, correlation between predicted and true values and normalized root mean squared error ($NRMSE$) were calculated. Both metrics were reported as a mean value and 95% confidence interval based on the metric distribution across all ten test folds. Since the patient-level splits lead to imbalanced test fold sizes, weighted means and weighted standard deviations were calculated.

### Symptom Severity and Alliance Prediction

As part of the machine-learning pipeline, we integrated a minimum redundancy maximum relevance feature selection algorithm (MRMR; Peng et al., 2005) via the python library *featurewiz* (Seshadri, 2023). We compared seven different regression-based machine-learning algorithms, most of which have been successfully employed for psychotherapy research before (Aafjes-van Doorn et al., 2021): (1) Elastic net regularization and variable selection (Elastic Net; Zou & Hastie, 2005) conducts both L1 and L2 regression regularization; (2) eXtreme Gradient Boosting (XGBoost; Chen & Guestrin, 2016) creates a series of decision trees that are trained sequentially for error correction, resulting in a final ensemble of trees; (3) Random Forest (RF; Breiman, 2001) also creates an ensemble of decision trees, which is aggregated to calculate the average of the predictions; (4) Support-Vector-Regression (SVR; Cortes & Vapnik, 1995) identifies a hyperplane that separates the data points into different classes by maximizing the distance between the hyperplane and the nearest data points from the classes; (5) Mixed Effects Random Forest (MERF; Hajjem et al., 2014) is an extension of RF allowing a random intercept for nested data (i.e., sessions nested in patients); (6) Gaussian Process Boosting (GPBoost; Sigrist, 2022) employs a boosting framework incorporating Gaussian Process regression and mixed effects modeling; (7) SuperLearner (van der Laan et al., 2007) is an algorithm that takes the predicted data from other algorithms as predictors and feeds them into a meta-algorithm that predicts the target variable. We chose an SVR algorithm as our meta-algorithm and used the predicted data from the previous algorithms (Elastic Net, XGBoost, RF, SVR, MERF, GPBoost) as predictors.

### Sensitivity Analyses

We calculated the following sensitivity analyses for the best model for the prediction of symptom severity and the best model for the prediction of alliance:

#### Random-Intercept-Only Model

 Since MERF and GPBoost both employed a random intercept, we decided to calculate an empty model with Random-Intercept-Only to assess how much of the prediction accuracy is due to the random intercept.

#### Model Without Random Intercept

 In addition to the Random-Intercept-Only-Model, we calculated a model only with Elastic Net, XGBoost, RF, SVR, and SuperLearner (= without a random intercept).

#### 50 Topics Model

 To assess the impact of the number of topics, we calculated a model with only 50 topics, leaving everything else the same.

#### LDA Model

 In order to compare BERTopic with a “classic” topic model, we used Python’s Gensim library (Rehurek & Sojka, 2010) to calculate an LDA model with 250 topics. For the pre-processing we used spaCy (Montani et al., 2023) for lemmatization and NLTK (Bird, Stefen, Loper, & Klein, 2009) for stopword removal.

#### Uni- and Bigram Model

 We calculated a model based on all uni- and bigrams in the transcripts after stopword removal and lemmatization. Using tf-idf weighting to highlight more important words, we yielded 400,000 + features each for patient and therapist speech. To reduce the number of features, we preselected the 500 best features by assessing the 500 best F-scores for the regression.

### Model Explanation

In order to assess feature importance, we used the SHAP (SHapley Additive exPlanations) package in python (Lundberg et al., 2020; Lundberg & Lee, 2017). Typically, machine learning models are very difficult to explain and unfortunately, often the most complex models tend to perform best (Holzinger et al., 2022). However, one method proposed in the literature to address this problem is the use of Shapley values. Shapley values were discovered by Shapley (1951) and were used to calculate the contribution of a player in an economic game and their respective payoff. Within the SHAP package, this approach is adapted to calculate the importance of each feature (Lundberg et al., 2020) by using SHAP values, which can be approximated for almost any machine learning algorithm. Further, we used the topic theme clustering to identify the importance of each theme as they correspond to the aggregated sum of absolute values of the associated topics. To account for positive and negative associations, theme importance was calculated separately for topics that were positively and negatively correlated with the outcome (e.g., 5 topics of a theme are associated with higher outcomes with a sum topic importance of 10% and 3 topics of a theme are associated with lower outcomes with a sum topic importance of 3%). This allowed us to identify not only the importance of each individual topic, but also the amount of negative and positive theme importance. Feature importance was calculated as the percent proportion of the absolute SHAP value of a topic or theme of the sum absolute SHAP value across all features.

## Results

### Themes and Their Assigned Topics

#### Patient Topics and Themes

In general, the topics were coherent and could be identified and clustered well. Of 250 topics, only 16 topics were put in the “incoherent” cluster, which in turn showed that 234 of 250 (93.6%) topics were meaningful. We identified 13 themes across all of the 250 topics in the qualitative analysis. In Table 1 we present all themes and two representative topics each. On average, 19.3 topics were associated with one theme. Also, almost all themes could be connected towards therapy in a sensible way (e.g., activities, income, family, health, …). Even though we did not aim to cluster similar themes for patients and therapists, we found that all patient themes were also present in the therapist themes.

**Table 1:** Themes within the patient topics

Theme	Number of topics	Topic name	Representative topic
Activities	19	Swimming	Swimming, water, swimming pool, going swimming, sea, beach, sauna, walking, waves, swim
		Planting	Flowers, garden, rose, plant, beautiful, flower, leaves, sun flowers, roses, weeds
Evaluations	29	Hot	Warm, hot, heat, very hot, warmth, sweaty, rather hot, felt warmth, warmer, tremble
		Small	Less, small, smaller, more less, very small, less more, lower, low, little less
Everyday life	35	Food	Eating, eat, eaten, hungry, cooking, bread, salad, cook, eats, breakfast
		Hair	Hair, hairdresser, at the hairdresser, cutting, cut, black hair, at
Income	9	Work	Work, job, go to work, occupation, worked, side job, go, workplace, job center
		Pension	Pension, pension insurance, pensioner, job center, applied, a third, pension procedure, receive pension, years, start at job center
Family	14	Children	Child, childhood, child child, small child, always, baby, which, small
		Marriage	Married, marriage, marry, man, years, wedding, at that time
Health	20	Hospital	Doctor, hospital, GP, clinic, at the, patient, name, said
		Depression	Depression, depressed, downcast, illness, compulsions, more depressed, depression group, went
Incoherent	16	–	Said, says, know, does, always, even, really, had
		–	Does, law, a friend does, criminology, highest score, know, score, dumplings, does any
Interpersonal	12	Friendship	Friend, friendship, friends, friendships, are friends, circle of friends, together
		Relationship	Relationship, partner, relationships, love, in love, loving, partnership, really, being in love, fully
Intrapersonal experience	17	Reflecting	Pondered, thought thought, thought about, thought, head, thought think, about, thoughts
		Dreaming	Dream, dreamt, dreaming, nightmares, caravan, about, dreamt about, dream about
Negative experiencing	28	Fear	Fears, fear fear, of, fear of, always fearful, little bit fear, fear of this, think
		Conflict	Fighting, fight, argue, duel, conflicts, always fighting, fought, gave
Positive experiencing	13	Fun	Funny, fun, laughed, laugh, witty, laugh laugh, joke, humor
		Gladness	Happy, pleased, glad glad, very glad, friendly, glad about, really glad, always glad
Scheduling	18	Next week	Next week, next, Friday, Monday, weekend, Saturday, Thursday, Wednesday, Sunday
		Hours	Hours, minutes, two hours, half an hour, half, ten minutes, ten, two, four hours
Various	21	Christmas	Christmas eve, Christmas day, Christmas market, Christmas celebration, holidays, first Christmas holiday, short Christmas, year, Christmas presents
		Colors	Colors, black, red, green, black white, blue, green, grey, yellow

*Notes.* Some topics have less words in English, because some words became redundant after the translation for this publication

#### Therapist Topics and Themes

Similar to the patient topics, therapist topics were coherent. Only 20 topics were clustered in the “incoherent” category, meaning that 230 (92.0%) were meaningful. We identified 15 themes across all of the 250 therapist topics in the qualitative analysis. In Table 2 we present all themes and two representative topics each. On average, 16.7 topics were associated with one theme. As pointed out above, the therapist themes consisted of all patient themes but included two additionally themes (“Psychotherapy content” and “Psychotherapy framework”) that seemed particularly relevant for the context of therapy.

**Table 2:** Themes within the therapist topics

Theme	Number of topics	Topic name	Representative topics
Activities	19	Music	Music, sing, concert, songs, choir, flute, orchestra, instrument, concerts
		Planting	Flowers, garden, rose, plant, beautiful, flower, leaves, sun flowers, roses, weeds
Everyday life	21	Eating	Eat, eaten, chocolate, hungry, eat eat, cake, bread, eats, eating behavior
		Washing	Washing, laundry, hands, washed, hand washing, washed hands, washing machine, washing laundry, washed hands washed
Evaluations	17	Importance	Important, important important, very important, more important, important point, very
		Understanding	Understandable, understood, understanding, wrong, lying, understand well, made mistake
Income	7	Work	Work, working, job, job center, work placement, occupation, go to work, worked
		Boss	Boss, superior, boss said, secretary, department head, boss exactly
Family	13	Father	Father, dad, father father, father’s, father okay, say father, situation father, said father, remember father
		Mother	Mother, mother mother, mum, mother said, actually mother, okay mother, told mother, tell mother, relationship mother
Health	9	Medication	Medication, drugs, pills, take, take pills, tja, chemo
		Blood pressure	Blood pressure, measure blood pressure, blood pressure monitor, measure, leave home, home, blood pressure monitor home, okay blood pressure
Incoherent	20	–	Say, good, perhaps, little, just, even, always, exactly, more
		–	Came, uhm exactly, since really, went, uhm, oh right, at that time, past, trigger
Intrapersonal experience	17	Feeling	Feeling, feeling feeling, feel, felt, sense, feel feeling, good feeling, feeling even
		Mood	Mood, mood swings, influence, better mood, swings, causes, thoughts influence, moods, okay mood
Interpersonal	17	Relationships	Relationship, relationships, partnership, partner, partherships, actually relationship, topic, relational ear, relationship pattern
		Talking	talk, discuss, speak, about, talk about, talked, talked about
Negative experiencing	25	Depression	Depression, depressed, symptoms, mood, disorder, antidepressant
		Panic disorder	Panic attack, panic, panic attacks, fear, panic disorder, patients, symptoms, panic patients, state of panic
Positive experiencing	13	Laughing	Laugh, laugh laugh, funny, witty, laugh about, joke, grin, light laughter
		Energy	Energy, accu, more energy, strength, power, recharge accu, recharge, use, energy energy
Psychotherapy content	9	Relaxation exercises	Exercise, exercises, training, relaxation, relax, relaxation exercises, cd, relaxed, muscles
		Safe place	Safety, protect, protection, safe, place, safe place, safety behavior, safer
Psychotherapy framework	20	Questionnaires	Questionnaire, questionnaires, give, give questionnaire, fill, fill questionnaire, get questionnaire, filled questionnaire, filled
		Diagnostics	Diagnosis, interview, diagnoses, diagnostic, conducted interview, diagnostic interview, final diagnostics
Scheduling	14	Today	Today, today today, working today, today work today, today works, today today today, must today, today ok, today gladly
		Vacation	Vacation, holidays, vacation vacation, two weeks vacation, weeks, week vacation, two weeks, next week vacation
Various	22	Age	Old, age, old old, born, older, birth, nineteen, life age
		Phone number	Number, phone number, mobile number, number, new number, office, number number, call, eight

*Notes.* Some topics have less words in English, because some words became redundant after the translation for this publication

### Outcome and Process Analysis

#### Current Session Symptom Severity

Correlation and NRMSE metrics can be obtained from Table 3. Since the Random-Intercept-Only model (*r* = .79, 95%-CI 0.76, 0.83) could not be improved by any model, we compared all models only without random intercept (without the learners with random intercept, i.e. without GPBoost and MERF). Since the patient topics performed better than therapist topics, all sensitivity analysis were conducted on patient topics.

**Table 3:** Accuracy metrics for current session symptom severity

Features	Split condition	$\varvec{r}$		NRMSE	
		Mean	95%-CI	Mean	95%-CI
250 patient topics	Random	0.45	0.40, 0.50	0.90	0.87, 0.92
50 patient topics	Random	0.41	0.35, 0.48	0.91	0.88, 0.94
LDA patient topics	Random	0.26	0.21, 0.31	0.96	0.95 0.97
Patient uni-bigrams	Random	0.78	0.76, 0.80	0.63	0.61, 0.66
250 patient topics	Patient-level	0.28	0.17, 0.37	1.02	0.85, 1.19
250 therapist topics	Random	0.27	0.19, 0.35	0.98	0.95, 1.01
250 therapist topics	Patient-level	0.01	− 0.07, 0.09	1.20	0.84, 1.56

##### Patient Topics

 After the elimination of missings, 535 sessions remained. The BERTopic patient topic model showed an average $r$ of 0.45 (95%-CI 0.40, 0.51) by selecting mostly RF learners. Using only 50 topics instead of 250 led to slightly worse performance (*r* = .41, 95%-CI 0.34, 0.48). An LDA model of 250 topics performed worse as well (*r* = .26, 95%-CI 0.21, 0.31), while the uni- and bigram model showed the best performance (*r* = .78, 95%-CI 0.76, 0.80).

In the patient-level splits condition, different learners were chosen with an average $r$ of 0.29 (95%-CI 0.19, 0.40) for the BERTopic model.

##### Therapist Topics

 In the random splits condition, RF was always selected (*N* = 543). An average $r$ of 0.27 (95%-CI 0.19, 0.35) was calculated. For patient-level splits, different learners were selected and the correlation was estimated at 0.01 (95%-CI − 0.07,0.09).

#### Therapeutic Alliance

Correlation and NRMSE metrics are displayed in Table 4. Since the Random-Intercept-Only model (*r* = .64, 95%-CI 0.55, 0.73) could not be improved by any model, we compared all models only without random intercept. Since the therapist topics performed best, sensitivity analyses were conducted on therapist topics.

**Table 4:** Accuracy metrics for therapeutic alliance

Features	Split condition	$\varvec{r}$		NRMSE	
		Mean	95%-CI	Mean	95%-CI
250 patient topics	Random	0.12	0.03, 0.21	1.02	0.99, 1.05
250 patient topics	Patient-level	0.05	− 0.01, 0.12	1.17	0.74, 1.62
250 therapist topics	Random	0.20	0.16, 0.24	0.99	0.97, 1.01
50 therapist topics	Random	0.17	0.10, 0.24	1.02	0.97, 1.07
LDA therapist topics	Random	0.15	0.06, 0.24	1.00	0.97, 1.04
Therapist uni-bigrams	Random	0.48	0.29, 0.61	0.88	0.81, 0.94
250 therapist topics	Patient-level	0.16	0.12, 0.21	1.12	1.03, 1.26

##### Patient Topics

 Sample size was 527. The test folds were calculated by different learners. For random splits, mean $r$ was 0.12 (95%-CI 0.03, 0.21) and for patient-level splits 0.05 (95%-CI − 0.01, 0.12).

##### Therapist Topics

 Altogether, 527 rows were selected. For random splits, 9 test folds were calculated by RF and one by Elastic Net with an average $r$ of 0.20 (95%-CI 0.16, 0.24). The 50 topics model had slightly lower accuracy (*r* = .17, 95%-CI 0.10, 0.24). The LDA model performed worse as well (*r* = .15, 95%-CI 0.06, 0.24) and the best results were obtained by the uni- and bigram model (*r* = .48, 95%-CI 0.29, 0.61).

For patient-level splits, RF was selected for seven test folds, SVR, Elastic Net, and SuperLearner each once once. Average $r$ was 0.16 (95%-CI 0.12, 0.21).

### Model Explanation

We decided to focus on the models with random splits as they showed superior performance. Importance was calculated as the proportion of topic or theme absolute SHAP value on the sum absolute SHAP value across all features.

#### Symptom Severity

##### Patient Topics

**Fig. 2:**
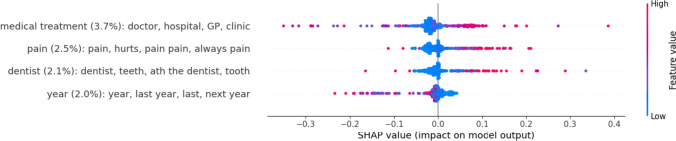
Patient topics with the highest impact on current session symptom severity

 In Fig. 2, all topics across the 2% threshold are shown with their respective percent SHAP value. The analysis allows for a deduction of the direction of the topic’s effect. If high feature value (= high topic frequency) is associated with positive SHAP values within the model, topic occurrence is associated with higher symptom severity. However, if high feature value is associated with negative SHAP values, the model interprets topic occurrence as associated with lower symptom severity. For example, all topics except for *year* are associated with higher symptom severity.

**Fig. 3:**
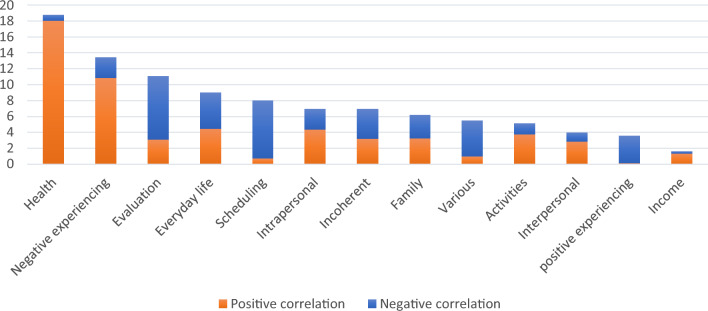
*A*ggregated importance of patient topics across themes (in %) for symptom severity

On the level of themes, regarding aggregated importance, especially *health* (18.7%) and *negative experiencing* (13.4%) were important themes for the model prediction (see Fig. 3). Themes that were especially associated with higher symptom severity were health, income, and negative experiencing, while scheduling, various, and positive experiencing were strongly associated with lower symptom load.

##### Therapist Topics

**Fig. 4:**
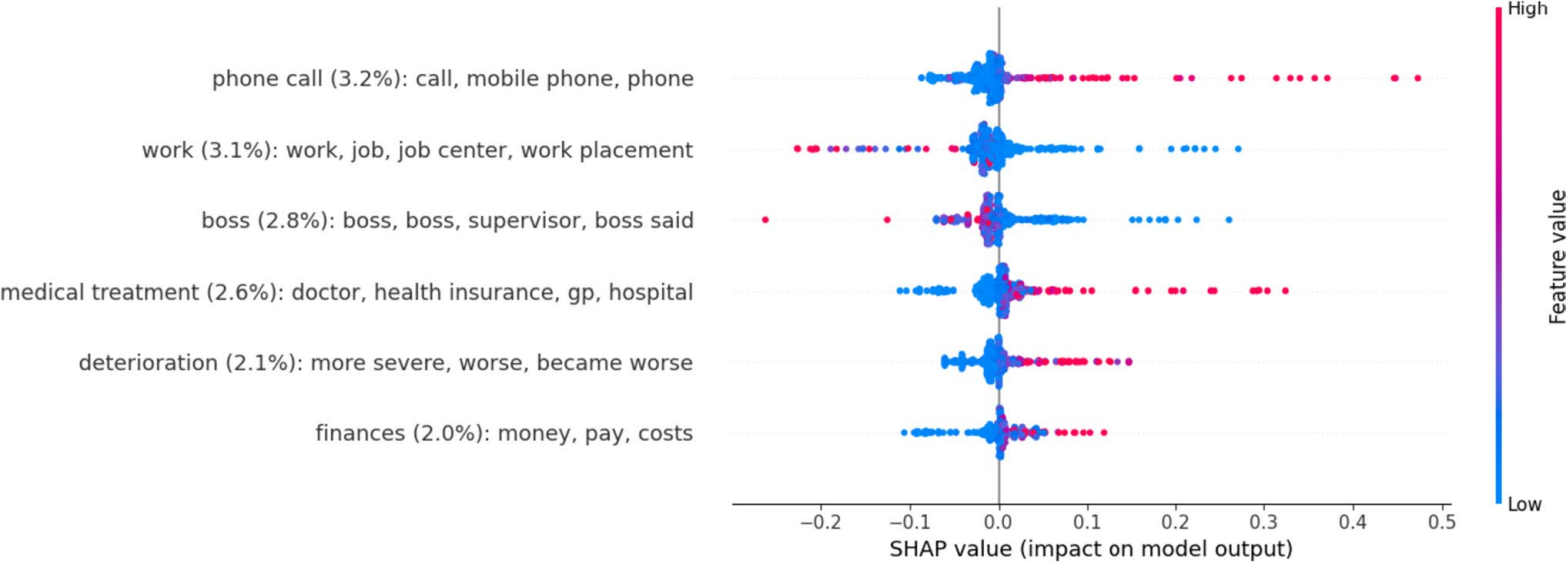
Therapist topics with the highest impact on symptom severity

**Fig. 5:**
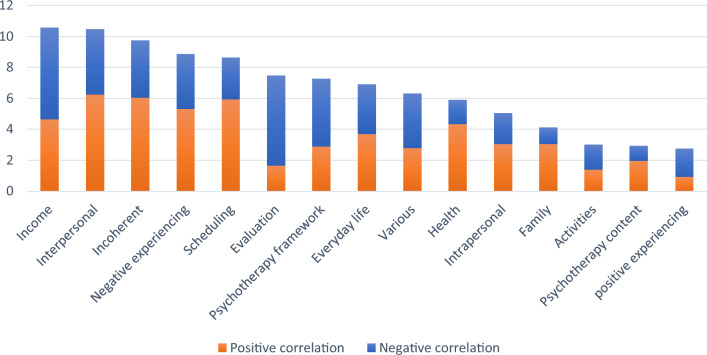
Aggregated importance of therapist topics across themes (in %) for symptom severity

Topics with the highest predictive value can be obtained from Fig. 4. The themes (see Fig. 5) with highest aggregated topic importance were *income* (13.9%) and *interpersonal* (11.3%). The themes *family* and *health* were associated with higher symptom severity while *evaluation* was associated with lower symptom severity.

#### Therapeutic Alliance

Due to the lower performance of the patient topic model, our analysis focused on the therapist topics.

##### Therapist Topics

**Fig. 6:**
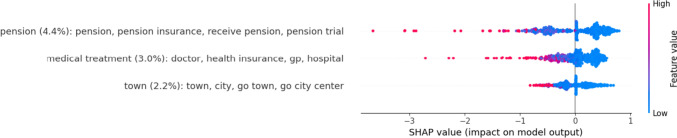
Therapist topics with the highest impact on alliance

**Fig. 7:**
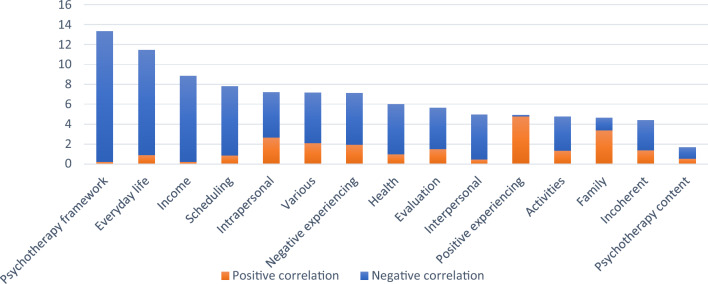
Aggregated importance of therapist topics across themes (in %) for alliance

 Regarding therapist topics, the topics *pension*, *medical treatment*, and *town* achieved importance over the 2% cut-off and were associated with lower alliance scores (see Fig. 6). Regarding theme importance (see Fig. 7), *psychotherapy framework* (13.3%), *everyday life* (11.4%), and *income* (8.9%) had the highest aggregated importance. Many themes were associated with lower alliance scores, especially psychotherapy framework, income, everyday life, and scheduling. Positive experiencing was associated with higher scores.

## Discussion

This study investigated the utility of transformer-based neural topic modeling (Grootendorst, 2022) for the prediction of symptom severity and alliance. Further, using an XAI framework, we identified topics and topic clusters (= themes) with the highest predictive power for the respective regression tasks both for patient and therapist topics.

Our topic model created 250 topics for both patients and therapists. These were qualitatively clustered into 13 patient, respective 15 therapist themes that were identical except for the two additional therapist themes. The 13 patient themes corresponded well to the seven themes identified by Atzil-Slonim et al. (2021), e.g., *positive experiencing* → positive experience, *negative experiencing* → negative experience, *family/interpersonal* → relationships, *health* → treatment/health, *everyday life* → everyday life, *various/incoherent* → miscellaneous while our remaining themes may be more specific (e.g., *activities, intrapersonal experience, income, evaluation, scheduling).*

Regarding the prediction of symptom severity, the best performing model was based on the patient topics with $r$=0.45 for random splits and $r$=0.29 for patient-level splits, resulting in good predictions only for random splits. Performance was slightly better than an LDA-based topic model with the same number of topics or a model with only 50 topics. However, the uni- and bigram model showed even better performance (*r* = .78).

Patient topics achieved better performance than therapist topics ($r$=0.27 for random splits), likely because symptom severity was rated by patients and not by the therapists. Especially the themes *health* and *negative experiencing* were predictors for higher symptom severity, which corresponds to findings that health problems predict depression severity (Iacovides & Siamouli, 2008; Iob et al., 2020) and that negative affect is associated with ruminative self-focus (Moberly & Watkins, 2008). Further, *income* correlated with symptom severity, possibly reflecting that lower socioeconomic status is associated with depression (Iob et al., 2020). *Positive experiencing* and *scheduling* correlated with less symptom severity, corresponding to findings that positive affect is inversely related to depression (Bean et al., 2022) and that higher activity levels are associated with less depression (Lewinsohn & Libet, 1972). It is interesting to note, that talking about specific activities (theme *activities*) is not associated with lower symptoms – only *scheduling* is.

Regarding the alliance, therapist topics achieved the best performance for random-splits (*r* = .20). Again, LDA-based topics (*r* = .15) and a model built on only 50 topics performed slightly worse with the best performance for the uni- and bigram model (*r* = .48). The superiority of therapist topics comes as a surprise, since patients rated the alliance in this study and not therapists. One explanation may lie in the fact that the therapist topics yielded two additional themes (*psychotherapy framework* and *psychotherapy content*) that were both relevant for the prediction (with *psychotherapy framework* as the best predictor) and not present in the patient topics. *Psychotherapy framework* and *income* were most clearly associated with lower alliance. For *income* this might be due to the sensitivity of the topic (White et al., 2021). The negative effects of *psychotherapy framework* seem puzzling. *Therapy framework* consists of procedures related to the therapy application (i.e., specialist report by the GP) and also to ROM. Looking at the individual topics, talk about either one of these aspects is associated with lower alliance scores, which could be the case when there are problems related to these procedures (e.g., the patient has forgotten to fill a questionnaire or to get the report). Only the theme *positive experiencing* was associated with higher alliance scores. This seems unsurprising, because positive emotions serve important social functions, such as bonding and goal motivation (Sels et al., 2021), which may both be reflected in a positive alliance. Looking across all themes, it can be concluded that a negative impact is far more likely than a positive impact. This illustrates a common finding in social psychology, namely that negative events have a stronger impact than positive events across a broad range of phenomena including interpersonal relations (Baumeister et al., 2001).

## Limitations and Future Directions

Several limitations of this analysis come to mind. First, we conducted very little hyperparameter tuning for BERTopic (varying document size for five and ten words and varying the number of topics between 150, 200, and 250 words). More tuning on other parameters could have been conducted (e.g., n-gram range, language model). Also, some topics were very homogeneous, because they consisted of different declinations of the same word (word flexion is common in German). This led to similar topics that were very homogeneous at the same time (e.g., topic *write1*: *written, write, wrote, writing* and topic *write2*: *write, write, write* [different German declinations]). Since BERTopic does not employ lemmatization like LDA, this has led to these homogeneous topics. However, BERTopic modeling still showed better performance than LDA, likely due to the superior embeddings. Second, even more specific themes could have been created which would have allowed for a more fine-grained analysis with better performance. Maybe that would also be a reason for the superior performance of the uni- and bigram model, which originally contained over 400,000 features. Third, the data set is still rather small, and it included on average only 4.5 transcripts per patient. Larger data sets would have allowed more stable results across different test sets and could possibly have led to better performance due to more training data. Fourth, model performance was not good enough to replace questionnaire data, and remained especially unsatisfactory for the alliance (*r* = .20). Since the uni- and bigram model showed better performance both for symptom severity and alliance, future research may be directed towards these features. Yet, the gains come with the cost of reduced interpretability, as uni- and bigrams are more difficult to comprehend than topics. However, considering vastly better performance, this could be addressed by clustering the features into more accessible superordinate categories.

Despite these limitations, we were able to use BERTopic, a state-of-the-art topic modeling approach to predict symptom severity and alliance. Topics were created separately for patient and therapist speech. We employed several machine-learning algorithms (Elastic Net, XGBoost, SVR/SVC, RF, MERF, GPBoost, and SuperLearner that competed in a nested cross-validation procedure for the best performance. This allowed us to use all available data as training data and to assess the performance across ten test sets. As a sensitivity analysis, we conducted the cross-validation with patient-level splits to evaluate model accuracy for new patients. Further, we compared the best results to a smaller topic model of only 50 topics^1^, an LDA-based topic model and a uni- and bigram model. Finally, even though we employed complex machine-learning algorithms, using an XAI framework allowed us to assess feature importance across all topic and themes.

Future directions may entail the use of topic synchrony features (e.g., Aafjes-van Doorn et al., 2020) for the prediction of the alliance. For example, differences in frequency of patient and therapist topics may be calculated as new features for better accuracy. Also, the approach could be used to provide feedback to therapists after each session by giving predictions of symptom severity and alliance, as well features that drive these predictions. For example, the therapist may receive a prediction of lower alliance scores for the last session because of a strong focus on income- and health-related themes. This could prompt the therapist to check in with the patient about their experience of the session. In general, topic modeling and similar NLP approaches could in the future be implemented as a feedback tool for a broad set of treatment-relevant variables (i.e., outcome, processes, and higher-order processes), supporting therapists to spot subtle negative trends and counter them early on (Lutz et al., 2022).

### Supplementary Information

Below is the link to the electronic supplementary material.

Supplementary material 1 (DOCX 28.6 kb)

## Footnotes

1 We also tested a combined model with patient and therapist topics, which did not improve the performance.
