# Supplementary material for: Measuring Alliance and Symptom Severity in Psychotherapy Transcripts Using Bert Topic Modeling
Source: Adm Policy Ment Health. 2024 Mar 29;51(4):509–24. doi: 10.1007/s10488-024-01356-4 (PMC11196307; doi:10.1007/s10488-024-01356-4)
Supplement: Supplementary file 1 — Supplementary material 1 (DOCX 28.6 kb) [file 10488_2024_1356_MOESM1_ESM.docx]

# **Declarations**

**Ethical Standards**

The authors assert that all procedures contributing to this work comply with the ethical standards of the relevant national and institutional committees on human experimentation and with the Helsinki Declaration of 1975, as revised in 2008.

**Contributor Roles Taxonomy (CRediT)**

**Christopher Lalk**: conceptualization, methodology, software, formal analysis, writing - original draft, project administration, **Tobias Steinbrenner**: software, data curation, writing – review & editing, **Weronika Kania**: data curation, **Alexander Popko**: software, writing – review & editing, **Robin Wester**: conceptualization, writing – review & editing, **Jana Schaffrath**: resources, **Steffen Eberhardt**: resources, **Brian Schwartz:** resources, writing – review and editing, **Wolfgang Lutz:** resources, writing - review and editing , **Julian Rubel**: conceptualization, methodology, resources, supervision, writing - review and editing

**External organizations**

Not applicable.

**Funding**

This work was supported by the German Research Foundation (DFG) under project no. 493169211.

**Conflict of interest.**

None declared.
